# Dapagliflozin-Associated Euglycemic Diabetic Ketoacidosis in a Patient Presenting with Acute Pancreatitis

**DOI:** 10.1155/2018/6450563

**Published:** 2018-08-07

**Authors:** Karun Badwal, Tooba Tariq, Diane Peirce

**Affiliations:** Western Michigan University Homer Stryker M.D. School of Medicine, USA

## Abstract

Sodium-glucose cotransporter 2 (SGLT-2) inhibitors are a class of medications used for glycemic control in type II diabetes mellitus. Their mechanism of action involves preventing resorption of glucose at the proximal kidney, thereby promoting glucosuria and weight loss. However, they have also been found to be associated with euglycemic diabetic ketoacidosis (euDKA). This case describes a 25-year-old male with a history of type II diabetes on metformin, sitagliptin, and dapagliflozin who was admitted with his third episode of pancreatitis secondary to hypertriglyceridemia. His home oral glycemic agents were continued as inpatient. Despite tight euglycemic control, the patient developed profound metabolic acidosis and was found to have an elevated beta-hydroxybutyrate level and normal lactic acid level. He was admitted into the intensive care unit and started on an insulin drip, and after resolution of his acidosis he was transitioned to basal insulin successfully. He was discharged with an insulin regimen while his oral glycemic agents were discontinued indefinitely. SGLT-2 inhibitors are associated with euDKA, most likely as a result of their non-insulin-dependent glucose clearance, hyperglucagonemia, and decreased ketone clearance. The aim of this case report is to inform the physician about the possibility of euDKA in a patient with type II diabetes on a SGLT-2 inhibitor presenting with an acute illness.

## 1. Introduction

Sodium-glucose cotransporter 2 inhibitors are a novel class of medications used for glycemic control in type II diabetes mellitus. They exert their effect by inhibiting SGLT-2 receptors in the kidney which are responsible for the resorption of glucose. This promotes glucosuria and in turn also decreases blood glucose levels [1].

This mechanism leads to the added benefit of weight loss by caloric loss in the urine, removing the energy source that drives excess adipose tissue formation resulting in improved insulin sensitivity [2, 3]. As dietary modifications and exercise are the mainstay of treatment in type II diabetes, SGLT-2 inhibitors can be used as monotherapy or as an adjunctive to other classes of oral glycemic agents. Common adverse effects reported in the literature are an increased incidence of genitourinary tract infections and orthostatic hypotension as a result of increased urinary frequency and volume. Recently, postmarketing surveillance has revealed an increased incidence of diabetic ketoacidosis in patients taking SGLT-2 inhibitor medications [4].

This case will illustrate a unique example of euglycemic diabetic ketoacidosis in a type II diabetic patient on a SGLT-2 inhibitor presenting with acute pancreatitis secondary to hypertriglyceridemia.

## 2. Case Presentation

**Figure 1:**
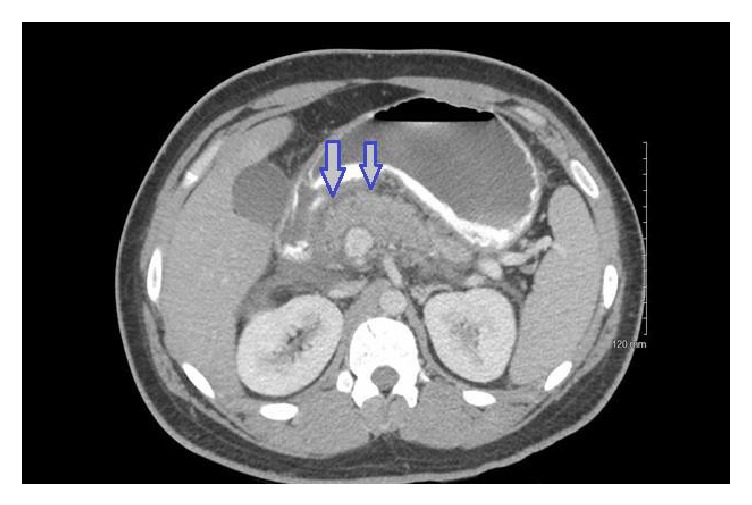
Peripancreatic inflammatory changes consistent with acute pancreatitis (arrows).

The patient is a 25-year-old gentleman who presented with a one-day history of abdominal pain, nausea, and emesis. He has had two episodes of pancreatitis in the past secondary to hypertriglyceridemia, with the last episode occurring three years ago. He also has type II diabetes controlled with dapagliflozin (SGLT-2 inhibitor), sitagliptin, and metformin. In the emergency department, the patient's initial labs showed a WBC of 23,000 cells/*µ*L, lipase of 2,530U/L, triglyceride level above 5,000mg/dL, bicarbonate 23mEq/L, and glucose 285mg/dL. His initial urinalysis and chest X-ray were unremarkable. A CT scan of his abdomen and pelvis with contrast was performed showing a large amount of peripancreatic inflammatory change consistent with acute pancreatitis (Figure 1). There was no evidence of cholelithiasis or cholecystitis, and the bile duct diameter was within normal limits. Based on these laboratory findings and imaging results, it was concluded that the patient had acute pancreatitis secondary to elevated triglycerides. He was admitted to the inpatient service and dapagliflozin, sitagliptin, and metformin were continued.

The patient was transitioned from nothing by mouth status on admission to a full-liquid diet on day 3 of hospital stay. By day 5, the lipase level trended down to 158U/L. His blood sugar remained consistently between 120mg/dl and 220mg/dl since admission. Despite maintaining tight euglycemic control, the patient developed profound metabolic acidosis with a gradual downward trend of his bicarbonate level from 23mEq/L to 5mEq/L and a high anion gap of 32 by day 5. This was accompanied by the acute development of tachypnea and tachycardia with a heart rate up to 130bpm. He was immediately started on an IV infusion drip of sodium bicarbonate. The beta-hydroxybutyrate level was 6.06mmol/L with a blood sugar of 161mg/dL and a lactic acid level of 1.5mmol/L. An arterial blood gas revealed a pH of 7.14 and pCO2 of 13mmHg. Although metformin was also continued, the normal lactic acid and elevated beta-hydroxybutyrate supported the diagnosis of DKA. It was concluded that the acidosis was secondary to diabetic ketosis induced by dapagliflozin. All oral glycemic agents were immediately discontinued, and he was transferred to the intensive care unit where he was started on an insulin drip. The nephrology service was consulted and by their recommendations the patient also underwent plasma exchange therapy for hypertriglyceridemia.

After being stabilized in the intensive care unit over the course of 24 hours, he was transferred to the general medical floor on an insulin drip and was transitioned to basal insulin. His diet was cautiously advanced in the setting of acute pancreatitis. Mealtime insulin coverage was added as the patient increased his oral intake. His blood sugars continued to remain well controlled between 120mg/dl and 200mg/dl while his insulin regimen was optimized according to his oral intake. He was discharged on an insulin regimen with insulin detemir and insulin lispro with the recommendation to stop all oral glycemic agents.

## 3. Discussion

SGLT receptors are a family of sodium glucose cotransporters that are primarily located at the brush border of the proximal convoluted tubules in the kidney [5]. SGLT-2 receptors are a high capacity, low affinity transporter that utilizes the sodium gradient to drive the reabsorption of approximately 90% of the filtered glucose in the S1 segment of the proximal renal tubule. The remaining glucose is reabsorbed by SGLT-1 receptors, which are placed more distally in the S3 segment of the proximal tubule and have a higher affinity but lower capacity for glucose [6]. The SGLT-2 inhibitor class of medications specifically inhibit the SGLT-2 transporters and therefore prevent the reabsorption of the majority of filtered glucose [4, 7, 8]. An added benefit of SGLT-2 inhibitors is the induction of modest weight loss by caloric loss of glucose in the urine leading to decreased visceral and subcutaneous adipose tissue and thereby further improving insulin sensitivity [2]. Finally, some of these agents have also been shown to have cardiovascular mortality benefits [9].

Canagliflozin, dapagliflozin, and empagliflozin are the three SGLT-2 inhibitors currently approved by the FDA for treatment of type II diabetes. However, since the approval of canagliflozin in March 2013, more than 70 cases of DKA have been reported. In May 2015, the FDA issued a warning about the risk of ketoacidosis with the use of SGLT-2 inhibitors [10–12]. Since then, further studies have investigated the incidence of SGLT-2 inhibitor-associated DKA. One study looking at the FDA Adverse Effect Reporting System database identified 7836 patients taking a SGLT-2 inhibitor, out of which 51 patients developed DKA with metabolic data. Of those 51 patients, 20 patients were type I diabetics, 25 patients were type II diabetics, and 6 patients were an unspecified type of diabetes. The study estimated a 7-fold increase in the incidence of DKA with patients on a SGLT-2 inhibitor when compared to patients on DPP-4 inhibitors with type II diabetes [13].

Diabetic ketoacidosis is a serious and potentially life-threatening complication of diabetes mellitus which occurs as a result of profound insulin deficiency. It is more commonly associated with poorly controlled type I diabetes as opposed to type II diabetes, in which case an added stress is required to trigger DKA such as infection or surgery [9]. Euglycemic DKA is an uncommon form of ketoacidosis which is characterized by metabolic acidosis with a pH <7.3 and a serum bicarbonate of <18mEq/L, ketosis, and a blood glucose level of <200 mg/dL [14].

The pathogenesis of DKA is well-known and involves low insulin levels triggering lipolysis and subsequent increased levels of free fatty acids in the blood which stimulates glucagon production. Glucagon promotes the oxidation of fatty acids and results in the production of ketone bodies, which are water-soluble acidic molecules directly responsible for ketoacidosis. Interestingly, the euDKA caused by SGLT-2 inhibitors follows a different mechanism of action. SGLT-2 inhibitors deplete the circulating glucose in the serum by promoting glucosuria, removing the stimulus for beta cells to secrete insulin. This in turn causes enhanced glucagon production by alpha cells in the pancreas. Furthermore, Bonner et al. demonstrated that both SGLT-1 and SGLT-2 cotransporters are also present on the alpha cells in the pancreas by confocal imaging analysis. Additionally, they found that the inhibition of SGLT-2 cotransporters by dapagliflozin in human islets was correlated with increased glucagon secretion by alpha cells [15]. Finally, there are studies suggesting that SGLT-2 inhibitors may decrease renal excretion of ketone bodies, therefore raising the level of ketone bodies in the blood. The end result of these combined mechanisms is hyperketonemia in the setting of euglycemia [9, 16]. Additionally, euDKA associated with SGLT-inhibitors causes twice the amount of renal glucose clearance compared to DKA [17].

At this time, SGLT-2 inhibitors are not approved by regulatory authorities for the treatment of type I diabetes. However, they are currently being used off-label due to their beneficial effects of weight reduction and maintenance of lower blood glucose levels in conjunction with insulin therapy [18, 19].

It is important to note that it is not common for individuals on a SGLT-2 inhibitor to develop euDKA. In a meta-analysis published by Burke et al. in 2017, the leading risk factors that predispose an individual on a SGLT-2 inhibitor to DKA include medication noncompliance, infection, major surgeries, and underlying autoimmune diabetes in patients previously diagnosed with T2DM [20]. In our patient, acute pancreatitis secondary to hypertriglyceridemia was thought to be the main driving force that led to the development of ketoacidosis. The patient was also not eating for the initial 48 hours of admission, possibly leading to a catabolic state with subsequent ketone body formation in the setting of a SGLT-2 inhibitor. This is supported by a randomized control trial study by Yabe et al. which randomized individuals on the SGLT-2 inhibitor luseogliflozin to diets of differing carbohydrate intake and found a higher incidence of ketoacidosis among individuals in the lower carbohydrate group [21].

SGLT-2 inhibitor-induced DKA is treated in a similar fashion as conventional DKA with the goal of driving the acidemia down with aggressive fluid resuscitation, insulin infusion, and close electrolyte monitoring. SGLT-2 inhibitors should be reinitiated only after consultation with an endocrinologist.

## 4. Conclusion

Historically, the majority of the cases of euDKA were missed due to presence of euglycemia on presentation [22]. Similarly, in our patient the diagnosis was delayed since his anion gap acidosis was initially attributed to starvation ketosis due to his nothing by mouth status in the setting of pancreatitis. However, after careful consideration a diagnosis of dapagliflozin-induced euDKA precipitated by acute pancreatitis was made. This case elaborates the fact that SGLT-2 inhibitors should be initiated by a clinician cautiously and only after adequately weighing the risks and benefits of treatment, particularly in those with type I diabetes. Patients should be instructed to check their serum and urine ketones in case they feel unwell even if they have a normal blood glucose level. Hospitalized patients (particularly those undergoing surgery or suffering from infectious/inflammatory process) who are on SGLT-2 inhibitors at home should be evaluated and closely monitored for the development of ketonemia or ketonuria during the hospital course.
